# Integrate Molecular Phenome and Polygenic Interaction to Detect the Genetic Risk of Ischemic Stroke

**DOI:** 10.3389/fcell.2020.00453

**Published:** 2020-06-24

**Authors:** Xiaoying Li, Weilin Shi, Ruyou Zhang, Shuang Zhang, Wenying Hou, Yingnan Wu, Rui Lu, Yanan Feng, Jiawei Tian, Litao Sun

**Affiliations:** ^1^Department of Ultrasound, The Second Affiliated Hospital of Harbin Medical University, Harbin, China; ^2^Department of Physical Diagnosis, The Fourth Affiliated Hospital of Heilongjiang University of Traditional Chinese Medicine, Harbin, China; ^3^Department of Neurosurgery, The Second Affiliated Hospital of Harbin Medical University, Harbin, China; ^4^Department of Ultrasound, Xuanwu Hospital Capital Medical University, Beijing, China; ^5^Department of Ultrasound, Shenzhen University General Hospital, Shenzhen, China

**Keywords:** ischemic stroke, polygenic interaction, molecular phenome, Interaction network analysis, molecular function

## Abstract

Ischemic stroke (IS) is one of the leading causes of death, and the genetic risk of which are continuously calculated and detected by association study of single nucleotide polymorphism (SNP) and the phenotype relations. However, the systematic assessment of IS risk still needs the accumulation of molecular phenotype and function from the level of omics. In this study, we integrated IS phenome, polygenic interaction gene expression and molecular function to screen the risk gene and molecular function. Then, we performed a case-control study including 507 cases and 503 controls to verify the genetic associated relationship among the candidate functional genes and the IS phenotype in a northern Chinese Han population. Mediation analysis revealed that the blood pressure, high density lipoprotein (HDL) and glucose mediated the potential effect of SOCS1, CD137, ALOX5AP, RNLS, and KALRN in IS, both for the functional analysis and genetic association. And the SNP-SNP interactions analysis by multifactor dimensionality reduction (MDR) approach also presented a combination effect of IS risk. The further interaction network and gene ontology (GO) enrichment analysis suggested that CD137 and KALRN functioning in inflammatory could play an expanded role during the pathogenesis and progression of IS. The present study opens a new avenue to evaluate the underlying mechanisms and biomarkers of IS through integrating multiple omics information.

## Introduction

Stroke is one of the three most common causes of death, and a major cause of adult disability in developed and developing countries, accounting for almost 6.5 million stroke deaths each year, and its global burden continues to grow (Endres et al., 2011; Bejot et al., 2016). The majority of global stroke burden is in low-income and middle-income countries. About 87% of all strokes are ischemic stroke (IS) and occurs when there is an obstruction in the blood vessel resulting in irreversible brain injury (Benjamin et al., 2019). Hence, there is a need to develop an effective prevention approach for stroke, which necessitates a better understanding of the underlying risk factors.

Omics data play a key role in evaluating the underlying mechanisms and predicting biomarkers. IS is a heterogeneous syndrome, many risk factors such as hyperlipidemia, hypertension, diabetes mellitus and inflammation have been confirmed as the important roles in the development of IS (O’Donnell et al., 2016; Beheshti et al., 2018). Evidence from studies of twins and family history demonstrates that genetics also influences the pathogenesis of IS and it should be responsible for a large part of IS risk (Dichgans and Markus, 2005; Traylor et al., 2012). More recently, the data from genome-wide association study (GWAS) have shown that dozens single nucleotide polymorphism (SNP) are related to IS (Matarin et al., 2007), however, these identified loci could explain only a small fraction of susceptibility. The genetic factor operates through a mechanism involving multiple genes, and is also affected by environmental factors (Dichgans and Markus, 2005), besides, the risk attributable to any individual variant has been modest. Furthermore, SNP and IS in a non-linear manner, the relevant risk factors have been frequently observed in the modulation effects of SNP on IS. Previous studies have focus on the single omics and research on SNP has been largely limited on single loci. Nevertheless, computational analysis provides a good way to understand the interactions between different factors that may contribute to IS occurrence. Some reports have shown that network analysis of SNP related dysregulated expression are associated with complicated diseases including IS. Importantly, fully understand the genetic factors in a multi-omics perspective helps to clarify the underlying molecular mechanisms contributing to IS. This study aimed to explore the combined effect of SNP from multiple genes and their interaction with risk factors in the development of IS. Furthermore, we also performed functional analyses of the selected genes at the transcriptome.

14 SNPs in RNLS, ALOX5AP, CD137, KALRN and SOCS1 were selected in the present study. The 5 genes involved in hypertension, inflammation, atherosclerosis and so on, and have been linked to IS in previous studies (Helgadottir et al., 2004; Zhang et al., 2013; Dang et al., 2015; Ma et al., 2016; Zhang et al., 2017). Including: rs10887800, rs2576178 and rs2296545 in the gene RNLS (renalase); rs4073259 in the gene ALOX5AP (arachidonate 5-lipoxygenase activating protein); rs17286604, rs7620580, rs11712619 and rs6438833 in the gene KALRN (kalirin RhoGEF kinase); rs243327, rs243330 and rs33932899 in the gene SOCS1 (suppressor of cytokine signaling 1); and rs161827, rs161818 and rs161810 in the gene CD137 (TNF receptor superfamily member 9). The relationship between SNP genotypes and IS risk factors were examined, we used mediation analysis to investigate the risk factors in the mediation effect on the association between SNP and the IS. And the case-control analysis of SNP-SNP interactions in the MDR approach was applied. Furthermore, the RNLS, ALOX5AP, CD137, KALRN and SOCS1 related mRNA interaction network was constructed and gene ontology (GO)functional annotation was implemented. Our study could provide theoretical foundation and reliably biomarkers for the mechanism involved in the pathogenesis of IS.

## Materials and Methods

### Case-Control Subjects and Clinical Characteristics

A total of 507 unrelated patients with IS from the northeastern Chinese Han population were consecutively recruited from the same center (Neurology Department, 2nd Affiliated Hospital of Harbin Medical University) and included in this study. All recruited patients were diagnosed by an experienced neurologist according to their clinical features and by auxiliary diagnosis, including computed tomography (CT) and/or magnetic resonance imaging (MRI) and routine laboratory tests. Subjects with infection, cerebrovascular malformation, autoimmune disease and history of takayasu arteritis were excluded from the study. In the case subjects, 311 subjects had hypertension, 112 subjects had diabetes and 68 subjects suffered from heart disease. Based on the Trial of Org 10172 in Acute Stroke Treatment (TOAST) classification the IS patients were further categorized into atherothrombosis subtype, lacunar infarction subtype and both atherothrombosis and lacunar infarction subtype(Adams et al., 1993).

The control group consisted of 503 unrelated gender-matched and geography-matched healthy individuals from the Health Medical Center, they were without stroke, hypertension, diabetes, heart disease, and other central nervous system disorders.

All of the cases and controls agreed with the ethics of the study and signed informed consent. The research was approved by the ethics committee of 2nd Affiliated Hospital of Harbin Medical University.

### Sequencing Genotyping of Selected SNP in Case-Control Samples

All the SNP genotyping experiments were performed by the Shanghai BioWing Applied Biotechnology Company^1^ using the ligase detection reaction (LDR) (Huang et al., 2008). The target DNA sequences were amplified using a multiplex PCR-based method. The primer DNA sequences are shown in Table 1.

**TABLE 1:** Primers of target genes used in the PCR.

Primer name	Sequence (5′–3′)		PCR length
rs10887800	GCACAAGGAACCCTGGTTTA	TGGCCCCTCTATTTCCTCTT	230
rs2576178	AGTGGCCGTTCAAGCATTAG	AAAAGCCTGGAAATGGTGTG	218
rs2296545	GCTCAGGGAGCTGAGGGTAT	TACCTTGCTGTGTGGGACAA	156
rs4073259	CCAAAGGCTTCACCTCTGAT	CGGCACATGAAAACAGCAC	248
rs17286604	CTTTTCCTCATGTGGAAGGC	CTTTGACGTGATAACCACCC	100
rs7620580	CACACTAAGCAGACAGTCCA	GGACCCAGAGCCTTTTTACA	99
rs11712619	GCAGCAAAGCAATTGGTAAC	CCACAAGGCACACAAATAATC	99
rs6438833	AGTGCTTTGCTGAGTGTACC	GGTAATTATAAACACAACAGC	101
rs243330	ATGGTGCATTCTCAGACGTG	GGGAAATCTATGAGGAAGGG	103
rs161818	CCTGACCCCTTCTTCCAAAT	ACTTGCTGTGGACCTATTGG	111
rs33932899	TCAGCCTTAGGACCCTCTC	TTCTAGCGAGTGTGCTTTGG	94
rs161810	GATCTCAAGGTCTGTCCATC	ATGGGCAAAGGGAGCAAAAG	102
rs161827	GGTTCCTAACAGATGGAAGC	TTCGAGATCCGATGGTTTCC	106
rs243327	TGGCCAGGGTGTGTAATCGT	TTCCCGACACATGGGTTAGA	102

Genomic DNA was extracted from EDTA-anticoagulated blood and the DNA samples were amplified in a 20 μl reaction mixture (containing 2 μl of 1 × buffer, 50 ng of genomic DNA, 0.6 μl Mg^2+^ (3.0 mmol/l), 2 μl of deoxynucleotide triphosphates (2.0 mmol/l), 2 μl of dNTP, 0.2 μl of 1U Taq Polymerase and 12.2 μl of ddH_2_O) by PCR. PCR amplification was applied to a thermal cycler in the Gene Amp PCR system 9600 (PERKIN ELMER). The primary information of the probes is shown in Table 2. The LDR was performed in a 10 μl reaction mixture: 1 μl of 1 × buffer, 1 μl of each probe mix 4 μl of ddH_2_O, 4 μl multi-PCR product and 0.05 μl of 2U of Taq DNA ligase. The fluorescent products of LDR were distinguished by an ABI sequencer 3730, and the results were analyzed with SHEsis and Genemapper software. The quality of genotyping was controlled using blinded blood duplicates.

**TABLE 2:** LDR target gene probe sequences.

Probe name	Sequence (5′–3′)	LDR length
rs10887800_modify	P-TGCAGCATGTTGGGACTCTCTTTTTTTTTTTTTTTTTT-FAM	
rs10887800_A	TTTTTTTTTTTTTTTTCATTCACTTCACTTTAAAAAGTT	77
rs10887800_G	TTTTTTTTTTTTTTTTTTCATTCACTTCACTTTAAAAAGTC	79
rs2576178_modify	P-GCGATACCACGGGCAACCTTTTTTTTTTTTTTTTTTTTTTTTTT-FAM	
rs2576178_C	TTTTTTTTTTTTTTTTTTTTAAAGTGGGAAGAAGAATTTACCG	87
rs2576178_T	TTTTTTTTTTTTTTTTTTTTTTAAAGTGGGAAGAAGAATTTACCA	89
rs2296545_modify	P-GACTCAGGTGGGTTGTCTAATTTTTTTTTTTTTTTTTTTTTTTTTTT-FAM	
rs2296545_C	TTTTTTTTTTTTTTTTTTTTTTTTGCTGTGTGGGACAAGGCTGAG	92
rs2296545_G	TTTTTTTTTTTTTTTTTTTTTTTTTTGCTGTGTGGGACAAGGCTGAC	94
rs4073259-R_modify-2	P-CTGGCTGGGGGTGACTCCAATTTTTTTTTTTTTTTTTTTTT-FAM	
rs4073259-R_T-2	TTTTTTTTTTTTTTTTTTGGCCTCTGCACGTGCTCTGCTCA	82
rs4073259-R_C-2	TTTTTTTTTTTTTTTTTTTTGGCCTCTGCACGTGCTCTGCTCG	84
rs17286604_modify	P-GTCCCCATCTCTCCATTGCCTTTTTTTTTTTTTTTTTT-FAM	
rs17286604_C	TTTTTTTTTTTTTTTTTTGTCTCATCTGCCTTGGAAATG	77
rs17286604_T	TTTTTTTTTTTTTTTTTTTTGTCTCATCTGCCTTGGAAATA	79
rs7620580_modify	P-GCACATGCAACCACTACCAATTTTTTTTTTTTTTTTTTTTT-FAM	
rs7620580_A	TTTTTTTTTTTTTTTTTTAGACAGTCCACACATGCACACAT	82
rs7620580_G	TTTTTTTTTTTTTTTTTTTTAGACAGTCCACACATGCACACAC	84
rs11712619_modify	P-TTCTTAATCACCTGTTACCATTTTTTTTTTTTTTTTTTTTTTTT-FAM	
rs11712619_C	TTTTTTTTTTTTTTTTTTTTACTTGACTAGATTTGAGTTCACG	87
rs11712619_T	TTTTTTTTTTTTTTTTTTTTTTACTTGACTAGATTTGAGTTCACA	89
rs6438833_modify	P-CTAAAACCAGCTGGTACACTTTTTTTTTTTTTTTTTTTTTTTTTTTT-FAM	
rs6438833_A	TTTTTTTTTTTTTTTTTTTTTTTTATTACCAATTTCAAGTGTTGT	92
rs6438833_T	TTTTTTTTTTTTTTTTTTTTTTTTTTATTACCAATTTCAAGTGTTGA	94
rs243330_modify	P-TCCTCTCCCCCGACCCCTTCTTTTTTTTTTTTTTTTTT-FAM	
rs243330_A	TTTTTTTTTTTTTTTTCCCCAAACCTGGTTTCCTAGCCT	77
rs243330_G	TTTTTTTTTTTTTTTTTTCCCCAAACCTGGTTTCCTAGCCC	79
rs161818_modify	P-GCCCCCCTTTGTTCAGTCCATTTTTTTTTTTTTTTTTTTT-FAM	
rs161818_A	TTTTTTTTTTTTTTTTTTTCTCGTCTTTTATTCCCGCTTTT	81
rs161818_G	TTTTTTTTTTTTTTTTTTTTTCTCGTCTTTTATTCCCGCTTTC	83
rs33932899_modify	P-CTGTTCAGAACCAAGTTAAATTTTTTTTTTTTTTTTTTTTTT-FAM	
rs33932899_C	TTTTTTTTTTTTTTTTTTTTCCTTAGGACCCTCTCCCCTGGAG	85
rs33932899_G	TTTTTTTTTTTTTTTTTTTTTTCCTTAGGACCCTCTCCCCTGGAC	87
rs161810_modify	P-CTCACCCCTTCTCAGTGCCCTTTTTTTTTTTTTTTTTTTTTTTT-FAM	
rs161810_A	TTTTTTTTTTTTTTTTTTTTTTGCAAAGGGAGCAAAAGTTCCAAT	89
rs161810_G	TTTTTTTTTTTTTTTTTTTTTTTTGCAAAGGGAGCAAAAGTTCCAAC	91
rs161827_modify	P-TTCCAAGGAAACCATCGGATTTTTTTTTTTTTTTTTTTTTTTTTTT-FAM	
rs161827_C	TTTTTTTTTTTTTTTTTTTTTTTTAATCCTGGAGTCATAACACTGCG	93
rs161827_T	TTTTTTTTTTTTTTTTTTTTTTTTTTAATCCTGGAGTCATAACACTGCA	95
rs243327_modify	P-GCCTCTGCCCCGGAATTCCTTTTTTTTTTTTTTTTTTTTTTTTTTTTT-FAM	
rs243327_C	TTTTTTTTTTTTTTTTTTTTTTTTTTCAGGGTGTGTAATCGTGAAACTG	97
rs243327_T	TTTTTTTTTTTTTTTTTTTTTTTTTTTTCAGGGTGTGTAATCGTGAAACTA	99

### Statistical Analysis

Hardy-Weinberg equilibrium (HWE) for genotypes were tested using the goodness of-fit χ^2^ test.

A mediation model was set up, with the risk factors (BMI, body mass index; systolic blood pressure, systolic BP; diastolic blood pressure, diastolic BP; glucose, Glu; TC, total cholesterol; TG, triglyceride; HDL, high density lipoprotein; LDL, low density lipoprotein) as a mediator, to test the direct and indirect effects of SNP (rs10887800, rs2576178, rs2296545, rs4073259, rs17286604, rs7620580, rs11712619, rs6438833, rs243327, rs243330, rs33932899, rs161827, rs161818, rs161810) on IS/IS subtypes. In this study, we used the R 3.5.3 to perform the mediation analysis. Mediation model and conclusion model used glm function. Mediation package was used in quantitative analysis of mediation effect. The model-based causal mediation analysis proceeds in two steps. First, the researcher specifies two statistical models, the mediator model for the conditional distribution of the mediator *Mi* given the treatment *Ti* and a set of the observed pre-treatment covariates *Xi* and the outcome model for the conditional distribution of the outcome *Yi* given *Ti*, *Mi*, and *Xi*. These models are fitted separately and then their fitted objects comprise the main inputs to the mediate function, which computes the estimated ACME and other quantities of interest under these models and the sequential ignorability assumption.

MDR analyses were implemented in the open-source MDR software package version 3.0.2^2^. This methodology can reveal high-order interactions among genes collaborating concerning a given phenotype, to determine multilocus genotype combinations associated with high or low risk of disease. The entropy-based clustering algorithm used by MDR sets a contingency table for k SNP and calculates case-control ratios for each of the possible multilocus genotypes. The prediction error of the model was evaluated by 10-folds cross-validation. The model with the smallest prediction error is selected as the final model, and the average prediction error of 10 tests is taken as the unbiased estimation of model-related prediction error. The hypothesis tests of the best model use the substitution test to evaluate the cross-validation consistency and the size of the prediction error estimates.

### Functional Enrichment Analysis

Functional enrichment analysis at the GO level was performed using the R pachage clusterProfiler. In our work, GO terms for “Biological Process” (GOTERM-BP-FAT) with a threshold of *p*-value < 0.05 were considered significant functional categories. Functional categories were visualized using DotPlot in R pachage clusterProfiler.

### Identification of mRNA-Target Interactions

The mRNA-miRNA interactions in our study were predicted with miRanda (version 3.3a) (John et al., 2004), which predicted miRNA targets based on sequence complementarity, conserved target sites and free energy of formation. The input 3′UTR sequences of mRNA from the Human (GRCh38) assembly were retrieved from the Ensembl database; the sequences of mature miRNA were downloaded from the miRBase database. To improve the reliability of miRNA target prediction, we used a maximum binding free energy of −20 kcal/mol for the miRNA target interaction predicted in miRanda.

### Identification of mRNA-mRNA Interactions

To identify competing mRNA-mRNA interactions, we used a hypergeometric test which enabled the evaluation of the significance of the shared miRNAs between each mRNA. We considered a *p*-value < 0.05 as statistically significant. The *p*-value was calculated as follows:

(1) $$P-v\,a\,l\,u\,e=1-\sum_{i=0}^{r-1}\frac{{∁}_{t}^{i}\,{∁}_{m-t}^{n-i}}{{∁}_{m}^{n}}$$

where m is the number of miRNAs in the network, and r represents the number of miRNAs shared between mRNAs. The number of miRNAs interacting with the RNLS, ALOX5AP, CD137, KALRN and SOCS1 and the other mRNAs is represented by *t* and *n*, respectively.

### Network Visualization and Topological Analysis

We used Cytoscape software (version 3.8.0) to construct and visualize the network in this study. Several topological properties such as the node degree, betweenness and closeness were analyzed using the built-in NetworkAnalyzer tool. The degree of a node is the number of edges that link to this node. Betweenness is a measure of the centrality of the node in a network, which is the number of shortest paths from each node to all others that pass through the node. The closeness of a node is the average length of the shortest path between the node and all other nodes in the network.

## Results

### Study Population for the Analysis of Ischemic Stroke Onset

All SNPs were found to be in Hardy-Weinberg equilibrium in all subjects (rs10887800: *p* = 0.9837; rs2576178: *p* = 0.9153; rs2296545: *p* = 0.9757; rs4073259: *p* = 0.7305; rs17286604: *p* = 0.8917; rs7620580: *p* = 0.87745; rs11712619: *p* = 0.9553; rs6438833: *p* = 0.9840; rs243327: *p* = 0.4587; rs243330: *P* = 0.5622; rs33932899: *p* = 0.9807; rs161827: *p* = 0.8320; rs161818: *p* = 0.8454; rs161810: *p* = 0.7297). The associations between each SNP and risk factors of IS were analyzed in correlation method. The rs2576178 genotype (CC + CT vs TT) was significantly related to drinking (*p* = 0.0392). The rs2296545 genotype (GG + CG vs CC) was revealed to be significantly correlated with smoking (*p* = 0.0267). The rs4073259 genotypes (AA vs AG vs GG) were significantly associated with HDL (*p* = 0.0013), and the GG + AG genotype was significantly with HDL (*p* = 0.0003) and blood glucose (*p* = 0.0375). The AA + AG genotype of rs4073259 was revealed to significantly with age (*p* = 0.024). The rs11712619 genotypes (CC vs CT) were significantly associated with HDL (*p* = 0.0032). The rs6438833 genotypes (AA vs AT vs TT) were associated with age (*p* = 0.0485) and systolic BP (*p* = 0.006). And the TT + AT genotype of rs6438833 was also associated with age (*p* = 0.0471) and systolic BP (*p* = 0.0099). The rs161818 genotypes (AA vs AG vs GG) were associated with systolic BP (*p* = 0.0377). And the GG + AG genotype of rs161818 was associated with systolic BP (*p* = 0.0152) and diastolic BP (*p* = 0.0242). The GG + CG genotype of rs33932899 was associated with drinking (*p* = 0.0365). The rs161810 genotypes (AA vs AG vs GG) were associated with systolic BP (*p* = 0.03) and diastolic BP (*p* = 0.0497). The GG + AG genotype of rs161810 was associated with systolic BP (*p* = 0.0149) and diastolic BP (*p* = 0.0214). The CC + CT genotype of rs161827 was associated with systolic BP (*p* = 0.0272).

The associations between the SNP and IS subtypes were also analyzed. The rs11712619 genotypes (CC vs TT) were associated with lacunar subtype of IS (0.0486). But no significant differences were found among the subtype of IS and the other SNP (Supplementary Table 1).

### The Mediating Effect of the Risk Factors on the Relation Between SNP and Ischemic Stroke

**FIGURE 1:**
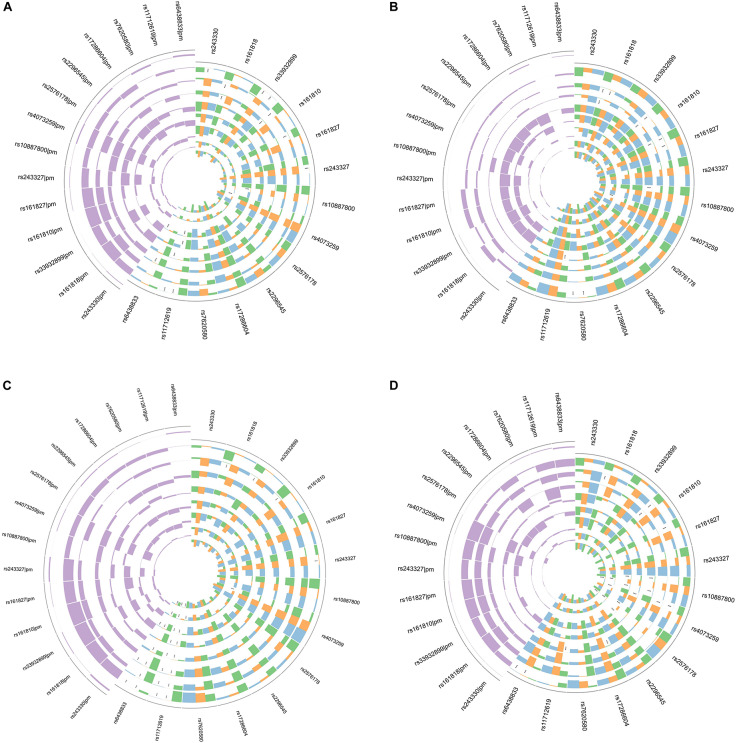
Circos plot showing the mediation model of the SNP, risk factors and IS **(A)**; SNP, risk factors and atherothrombosis subtype of IS **(B)**; SNP, risk factors and lacunar subtype of IS **(C)**; SNP, risk factors and combination subtype of IS **(D)**. Purple in the upper left corner showing Prop Mediated (pm). The outer layer to the inner of ring were BMI, systolic BP, diastolic BP, blood glucose, TC, TG, HDL, LDL, respectively. Green indicate mediated effect *p*-value; orange indicate direct effect *p*-value; blue indicate total *p*-value. ***p < 0.05.

In gene-risk factors-IS pathways the mediation models were set up, with the risk factors of the IS as a mediator, to test the direct and indirect effects of SNP on IS, the results are illustrated in Figure 1A. We found significant mediating effects in the systolic BP as mediator, from rs161818 (*p* = 0.014), rs161810 (*p* = 0.012) and rs161827 (*p* = 0.04) to IS, respectively, but the direct effect was no significant. In addition, the diastolic BP as mediator we found significant mediating effects, from rs161810 (*p* = 0.028), rs161818 (*p* = 0.038) to IS, respectively, but we did not find any significant direct effects. From rs4073259 (*p* = 0.002) to IS, there was a significant mediating effects in the HDL as mediator, the direct effects were no significant effects. The results suggest that the significant mediating effects are all complete mediating effects.

### The Mediating Effect of the Risk Factors on the Relation Between SNP and Ischemic Stroke Subtypes

To test the direct and indirect effects of SNP on IS subtypes, the mediation analysis was also performed in gene-risk factors-IS subtypes pathways. In gene-risk factors-atherothrombosis subtype of IS path models the results are showed in Figure 1B. In rs161818 on atherothrombosis subtype of IS path model, the mediating effect was significant from rs161818 to atherothrombosis IS through systolic BP (*p* = 0.028), and the direct effect also significant (*p* = 0.046), mediating proportion is 49.0%. In rs161827 on atherothrombosis subtype of IS path model, the mediating effect was significant from rs161827 to atherothrombosis IS through systolic BP (*p* = 0.04), and the direct effect also significant (*p* = 0.026), mediating proportion is 45.4%. We found significant mediating effects in the systolic BP as mediator, from rs161810 (*p* = 0.012) to atherothrombosis IS, and the direct effect were not significant. The diastolic BP as mediator we found significant mediating effects, from rs161818 (*p* = 0.032), rs161810 (*p* = 0.012), rs161827 (*p* = 0.046) to atherothrombosis IS, respectively, but we did not find any significant direct effects. The blood glucose as mediator we found significant mediating effects, from rs10887800 (*p* = 0.004), rs11712619 (*p* < 0.0001) to atherothrombosis IS, but the direct effect was no significant effect. We found significant mediating effects in the TG as mediator, from rs243327 (*p* = 0.002), rs243330 (*p* = 0.004) and rs33932899 (*p* = 0.004) to atherothrombosis IS, respectively, the direct effects were no significant effects. We found significant mediating effects in the HDL as mediator, from rs4073259 (*p* < 0.0001) to atherothrombosis IS, the direct effects were no significant effects.

In gene-risk factors-lacunar subtype of IS path models the results are showed in Figure 1C. We found significant mediating effects in the systolic BP as mediator, from rs161818 (*p* = 0.044), rs161810 (*p* = 0.038), rs33932899 (*p* = 0.036) to lacunar IS, and the direct effect were not significant. We found significant mediating effects in the HDL as mediator, from rs4073259 (*p* = 0.004), rs11712619 (*p* = 0.04) to lacunar IS, the direct effects were no significant effects.

In gene-risk factors-combination subtype of IS path models the results are showed in Figure 1D. We found significant mediating effects in the systolic BP as mediator, from rs161818 (*p* = 0.042), rs161810 (*p* = 0.038), rs161827(*p* = 0.04), rs4073259 (*p* = 0.042) to combination subtype of IS, but the direct effects were not significant. The diastolic BP as mediator we found significant mediating effects, from the rs161810 (*p* = 0.042), rs4073259 (*p* = 0.046) to combination subtype of IS, respectively, but we did not find any significant direct effects. The blood glucose as mediator we found significant mediating effects, from rs10887800 (*p* = 0.042), rs11712619 (*p* = 0.002), rs17286604 (*p* = 0.046) to combination subtype of IS, but the direct effect was no significant effect. We found significant mediating effects in the TC as mediator, from rs4073259 (*p* = 0.012) to combination subtype of IS, the direct effect was not significant. The TG as mediator we found significant mediating effects, from rs10887800 (*p* = 0.036), rs2576178 (*p* = 0.012) to combination subtype of IS, respectively, but we did not find any significant direct effects. We found significant mediating effects in the HDL as mediator, from rs4073259 (*p* < 0.0001) to combination subtype of IS, the direct effect was not significant.

### SNP-SNP Interactions by MDR Analysis With the Risk of Ischemic Stroke

**FIGURE 2:**
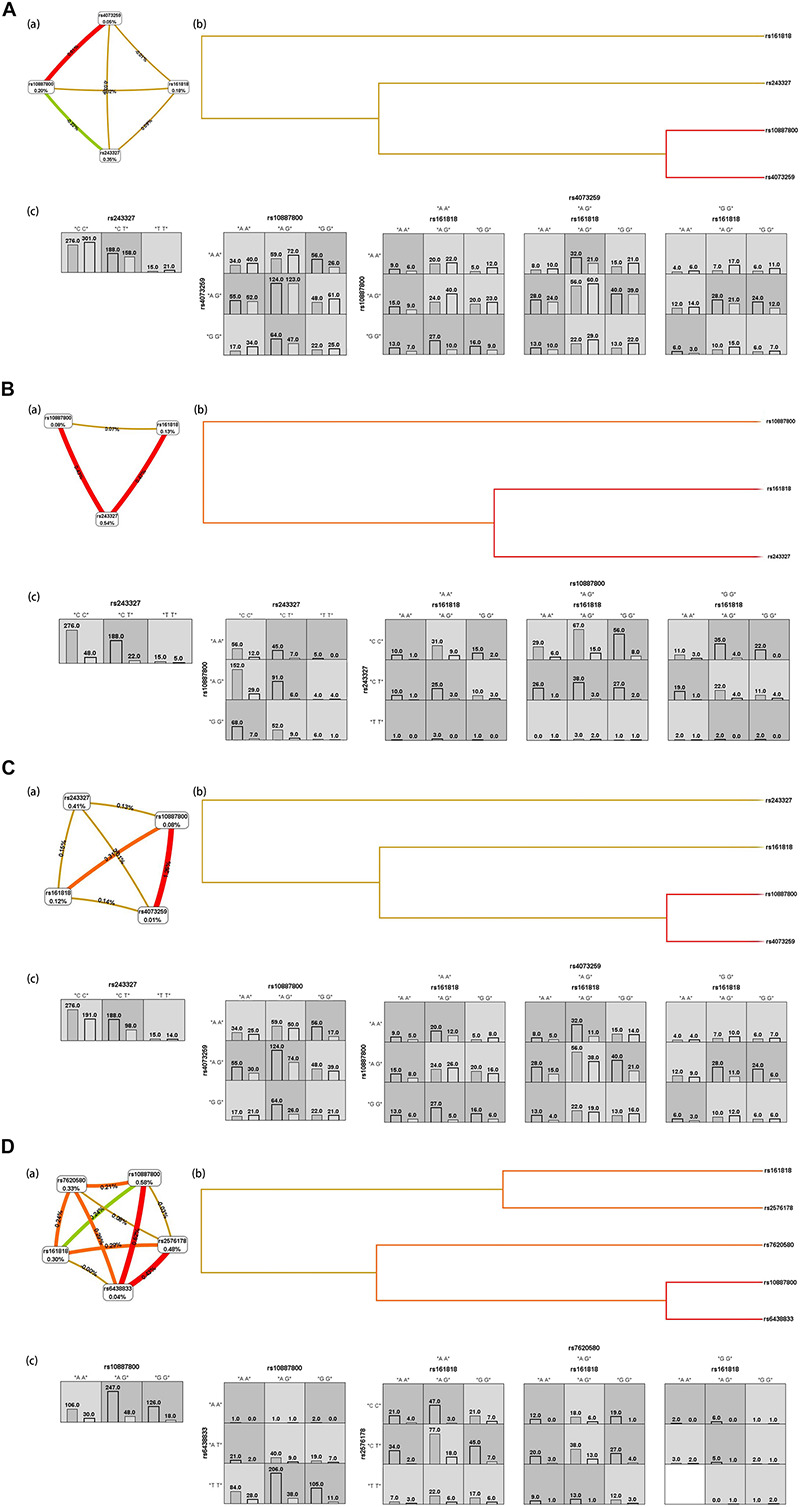
IS **(A)**, atherothrombosis subtype of IS **(B)**, lacunar subtype of IS **(C)**, combination subtype of IS **(D)** results from MDR analysis. **(a)** interaction graph, for each SNP is reported in per cent the value of Information Gain (IG), while numbers in the connections indicate the entropy-based IG for the SNP pairs. Red bar and orange bar indicate the high-level synergies on the phenotype, while the brown indicate a medium-level interaction, green and blue connections with negative IG values indicate redundancy or lack of synergistic interactions between the markers. **(b)** shows the interaction dendrogram. Histograms in **(c)** reports the distributions of controls (left bars) and cases (right bars) genotype combinations of SNP. Dark-shaded cells are considered “high-risk” while light-shaded cells are considered “low risk.” White cells indicate no subjects with those genotype combinations that were observed in the data set.

Figure 2A shows the interaction graph (a), interaction dendrogram (b) and histograms (c) for the whole genotypic data set, as resulting from MDR analysis. In this way, we explored whether there are significant SNP-SNP interactions between different SNP belonging to different genes in this article. A significant interaction model was found among rs161818, rs10887800 and rs4073259, and driving the high-risk combinations for IS (*p* < 0.0001, OR = 1.9893, 95%CI = [1.5355–2.5773]). We found another significant epistasis between rs10887800 and rs4073259 (*p* = 0.0008, OR = 1.5539, 95%CI = [1.2013, 2.0101]). And the rs243327 has the largest univariate effect (0.35% of explained entropy in the network) as the high-risk combinations for IS (*p* = 0.0412, OR = 1.3166, 95%CI = [1.0108, 1.715]).

### Combined Effects of the Associated SNP With the Risk of Ischemic Stroke Subtype

We then further explore the epistatic interactions between different SNP in regard to IS subtype risk. The rs161818, rs243327, and rs10887800 (*p* < 0.0001, OR = 2.9428, 95%CI = [1.7676, 4.8995]) performed a significant interaction model for atherothrombosis subtype of IS risk. Another significant interaction model for atherothrombosis subtype of IS risk we found is between rs243327 and rs10887800 (*p* = 0.0055, OR = 2.1287, 95%CI = [1.2371, 3.6628]), Figure 2B. For lacunar IS, we found two significant risk interaction model, they are rs10887800, rs4073259 (*p* = 0.0001, OR = 1.7628, 95%CI = [1.3171, 2.3593]) and rs161818, rs10887800, rs4073259 (*p* < 0.0001, OR = 2.1871, 95%CI = [1.6304, 2.9339]), respectively (Figure 2C). Figure 2D display the SNP-SNP interactions affection on combination subtype of IS, the SNP-SNP combinations jointly explain the rs10887800 SNP having the largest univariate effect (0.58% of explained entropy in the network). In this sub-data set, two meaningful SNP-SNP interaction models were found: one between rs10887800 and rs6438833 (*p* = 0.0014, OR = 2.0527, 95%CI = [1.3142, 3.2061]), and another among rs161818, rs2576178 and rs7620580 (*p* < 0.0001, OR = 3.034, 95%CI [1.8776, 4.9025]), all as the high-risk combinations for combination subtype of IS.

### GO Functional Annotation and Topological Analysis of the mRNA Interaction Networks

**FIGURE 3:**
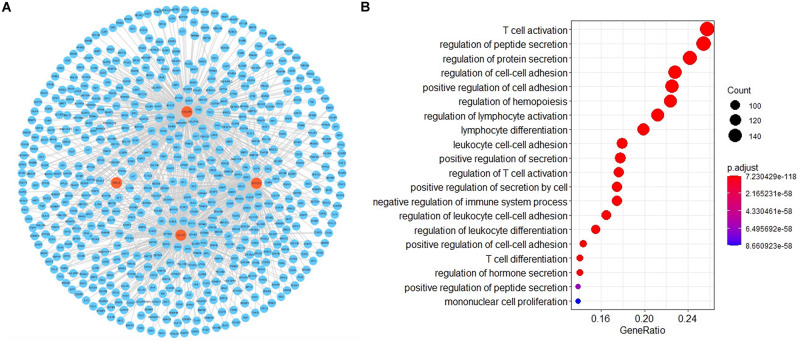
The layout of mRNA-mRNA network. The view of the mRNA-mRNA network **(A)**. The GO enrichment analysis of the network **(B)**.

To investigate the potential functional implication of RNLS, ALOX5AP, CD137, KALRN and SOCS1 in IS, we performed GO functional enrichment analysis for the five genes. We extracted the mRNAs related to the function of the five genes based on GO biological process terms. We identified putative miRNA-mRNA interactions using the miRanda software. We next used miRNA-mRNA paires to construct the miRNA-mRNA network, which included 107 miRNAs, 1476 mRNAs and 14678 interaction pairs. We observed a power-law distribution with a slope of −1.082 and an *R*-squared value of 0.793. This signifyed that the network displayed scale-free characteristics typical of a biological network rather than a random network. To identify miRNA-mediated mRNA-mRNA interactions, we used a hypergeometric test to investigate the potential interaction between mRNA pairs. We defined mRNA-mRNA competing pairs if they shared the same miRNA and had a *p*-value < 0.05. Then we obtained the RNLS, ALOX5AP, CD137, KALRN, and SOCS1 associated 3463 mRNA-mRNA pairs and constructed mRNA-mRNA interaction network (Figure 3A). We calculated three topological properties of the mRNA-mRNA network: the node degree, betweenness centrality and closeness centrality. Then, we ranked the topological features of all nodes and found that KALRN and CD137 were common nodes that were present in the top five of each index described above (Table 3). To explore the potential functional implications of KALRN and CD137, we performed functional enrichment analysis of GO for the mRNAs in the network (Figure 3B). These genes were enriched to the inflammation relevant terms: such as T cell activation, regulation of cell-cell adhesion, regulation of lymphocyte activation and so on.

**TABLE 3:** The top five mRNA with largest degree, betweenness and closeness in mRNA-mRNA network.

Top degree	Top betweenness	Top closeness
KALRN	KALRN	KALRN
CD137	PAX1	CD137
SOCS1	CD137	SOCS1
RNLS	ACE	RNLS
PAX1	ADGRG1	PAX1

## Discussion

IS is result of a reduction of blood flow and oxygen to the brain, due to occlusion the cerebral artery by embolus or a thrombus produces (Musuka et al., 2015; Hankey, 2017). The identification of genes and molecules that are related to IS occurrence is important for our understanding and control of the disease. With a substantial genetic component, the heritability of IS ranges from 16 to 40%(Bevan et al., 2012), GWAS and several case-control association studies revealed the contributory role of several variants belonging to different genes on IS and their specific subtypes, such as protein kinase C eta (PRKCH), T cell immunoglobulin and mucin domain containing 4 (TIMD4), cyclin dependent kinase inhibitor (CDKN) and arachidonate 5-lipoxygenase activating protein (ALOX5AP) (Wu et al., 2009; Traylor et al., 2012; Larsson et al., 2017; Liu et al., 2017; Khounphinith et al., 2019). Nevertheless, such association study is unable to explain the biological complexity of IS, which a multifactorial disease with a number of components being involved in its pathological mechanism, including numerous environmental and genetic risk factors. In this study, a multidimensional network was constructed to provide new insights on the mechanism underlying the progression of IS.

Although the relationships among gene, risk factors and IS have been extensively investigated, there is lack of a united gene-risk factors-IS model to account for the modulation of SNP on the contribution of IS. In this study, we used mediation analysis to investigate gene-risk factors-IS pathways to explain how SNP selected in this article polymorphism affects Is and IS subtypes via modulating the risk factors. In rs161818-systolic BP-atherothrombosis subtype of IS and rs161827-systolic BP-atherothrombosis subtype of IS pathways, the rs161818 and rs161827 has an effect on atherothrombosis subtype of IS directly, and also affect atherothrombosis subtype of IS through the mediating effect of systolic BP. And then, in other pathways of the study, SNP affect IS or IS subtypes through the mediating effect of risk factors. For instance, mediation analysis revealed that the blood pressure mediated the association between rs161818 (CD137), rs161810 (CD137), rs161827 (CD137) and IS/atherothrombosis subtype of IS; between rs161818 (CD137), rs161810 (CD137), rs33932899 (SOCS1) and lacunar IS subtype; between rs161818 (CD137), rs161810 (CD137), rs161827 (CD137), rs4073259 (ALOX5AP) and combination subtype of IS. And interestingly, the HDL mediated the effect of rs4073259 polymorphisms not only on IS, but on all IS subtypes in this study. The blood glucose mediated the effect of rs10887800 (RNLS), rs11712619 (KALRN) on atherothrombosis subtype of IS/combination subtype of IS. Concurrently, the blood glucose mediated the effect of rs17286604 (KALRN) on combination subtype of IS also. And then according to the MDR analysis, our findings revealed significant interactions between rs161818 (CD137), rs10887800 (RNLS) and rs4073259 (ALOX5AP) in the affection of outcome, increasing the risk of IS and lacunar IS subtype. For atherothrombosis subtype of IS, the significant interactions between rs161818 (CD137), rs243327 (SOCS1), rs10887800 (RNLS) were observed in the degree of risk. Another interaction model we found among rs161818 (CD137), rs2576178 (RNLS) and rs7620580 (KALRN) that increased the combination subtype of IS risk.

Hypertension is widely recognized as the most important modifiable risk factor for all types of stroke, the incidence of stroke increases proportionally with both systolic and diastolic pressure, increasing the relative risk 3.1-fold for men and 2.9-fold for women (Kannel et al., 1981; Cipolla et al., 2018). Hypertension acts in stroke through various mechanisms, such as affecting vascular smooth muscle cells (VSMCs), endothelial dysfunction, oxidative stress, changes in cerebral blood flow, and inflammation (Shekhar et al., 2017; Shekhar et al., 2018). In the current investigation, we used a mediation model and revealed that the blood pressure might mediate the modulation of SOCS1 and CD137 polymorphism on the IS. This gene-blood pressure-IS pathway may help us to better understand the IS mechanism. The systolic BP mediated the effect of rs161818 (CD137), rs161810 (CD137) and rs161827 (CD137) polymorphisms on the IS and atherothrombosis subtype of IS. Diastole BP mediated the effect of rs161818 (CD137) and rs161810 (CD137) polymorphisms on the IS. The diastole BP mediated the effect of rs161818 (CD137), rs161810 (CD137) and rs161827 (CD137) on the atherothrombosis subtype of IS. The systolic BP mediated the effect of rs161818 (CD137), rs161810 (CD137) and rs33932899 (SOCS1) on lacunar IS subtype. SOCS1 is expressed by immune cells, SOCS1 protein modulated inflammatory signaling (Metcalf et al., 2002; Davey et al., 2006), and it has been shown to be associated with hypertension via the JAK-STAT pathway (Ortiz-Munoz et al., 2010; Satou et al., 2012). CD137 is a member of TNF receptor superfamily, expressed primarily on activated T cells, natural killer (NK) cells, resting monocytes, and dendritic cells (DCs), CD137L as the ligand for CD137 is mainly expressed on DCs, B cells and macrophages (Watts, 2005; Shao and Schwarz, 2011). Recent studies suggest that CD137/CD137L interactions may activate NF-κB to secrete and elevate transcription for IL-6 and tumor necrosis factor α (TNFα), are critical for initiating and modulating antitumor immune responses (Kim et al., 2011). Increasing evidence have suggested that immune cells, such as TNF-α and IL-6, plays an important role in the blood pressure modulation, particularly in angiotensin II induced hypertension (Lee et al., 2006; Harrison et al., 2010; Harrison et al., 2011). Our findings also suggest that BP mediated the effect of SOCS1 and CD137 SNP on IS and atherothrombosis, lacunar IS subtype. The two genes may through an inflammatory mechanism interact with BP that is indirectly associated with IS.

Lipoprotein is widely recognized as the most important correlated factor for IS (Fuentes et al., 2009; Lapergue et al., 2010). In previous studies, the results from the investigations of the relationship between high levels of lipoprotein and IS are conflicting (Amarenco et al., 2008; Chei et al., 2013). This may be because lipoprotein is involved in the occurrence of IS through some mechanisms rather than directly in the pathophysiological process of IS. In the present study, a mediation model of gene-lipoprotein-IS pathway were measured. Interestingly, HDL mediated the effect of rs4073259 not only on IS, but on IS all subtypes in this study. ALOX5AP with 5-lipoxygenase (5-LO) is required for leukotriene synthesis, previous research also showed that ALOX5AP regulated the oxLDL-induced inflammation (Silva et al., 2009). HDL plays an anti-inflammatory role maybe involved in TNF-α-induced adhesion and NF-κB pathway (Zhu and Parks, 2012). The results from our data, HDL might mediate the inflammatory response that involved in IS and IS subtypes.

Hyperglycemia is common in patients with IS and is associated with worse outcomes (Zheng and Zhao, 2020). It is also well known that severe or long-term hypoglycemia can result in permanent brain damage. In our study, the glucose mediated the effect of rs10887800 (RNLS) and rs11712619 (KALRN) on the atherothrombosis subtype of IS and combination subtype of IS. In addition, the glucose mediated the effect of rs17286604 (KALRN) on combination subtype of IS. RNLS is a monoamine oxidase secreted by kidney and involved in the oxidative degradation of circulating catecholamines. Several RNLS SNPs have been linked to common human diseases, such as hypertension and diabetes. From literature suggest, RNLS involved in blood pressure regulation and endothelial dysfunction (Zbroch et al., 2012). KALRN genetic variations may lead to endothelial dysfunction and atherosclerosis through influence on the Rac-1 signaling pathway, which is associated with Type 2 diabetic(Mofarrah et al., 2016).

SNP-based approaches for identifying interaction may have no marginal effects to the trait but have strong interaction effects with other SNP (He and Lin, 2011). Furthermore, these approaches have limitations and cannot fully interpret in a viewpoint of SNP interactions (Li et al., 2015). SNP-SNP interaction analysis provide new insights into the genetic bases of complex diseases, and as an important way can portray the non-linearities in the relationship between genotype combinations and trait (Fang and Chiu, 2012; Ma et al., 2013). To fully understand the impact of the SNP-SNP crosstalk on IS, we used the MDR analysis by a classical case-control approach. The analysis showed that CD137, RNLS, and ALOX5AP significantly increased risk of IS and lacunar IS subtype. Besides, CD137, SOCS1, and RNLS were observed increased the degree of risk in atherothrombosis subtype of IS, and CD137, RNLS, and KALRN increased the combination subtype of IS risk. To explore the biological impact in IS of the RNLS, ALOX5AP, CD137, KALRN, and SOCS1, we performed GO enrichment analysis for the genes and found the enriched terms related genes. Then we used mRNA-miRNA interaction data predicted by the miRanda to construct a mRNA-miRNA network. Based on miRNA sponge, we next obtained the mRNA-mRNA interaction pairs using the hypergeometric test. GO enrichment analysis for the neighbor genes of RNLS, ALOX5AP, CD137, KALRN, and SOCS1 showed that those genes are involved T cell activation, regulation of cell-cell adhesion, regulation of lymphocyte activation. These biological processes are associated with the inflammation. As previously reports described, inflammation plays a major role in IS pathomechanism. Topological analysis of the mRNA-mRNA network, we found that CD137 and KALRN were common nodes in the top five of each index. Therefore, it is reasonable to hypothesize that CD137 and KALRN to regulate inflammatory mechanism during the pathogenesis and progression of IS.

In conclusion, this study opens a new avenue to evaluate underlying mechanisms and biomarkers of IS through integrating multiple omics data. We identified a gene-risk factors-IS/IS subtypes pathway to explain how the SNP in this study affects IS and IS subtypes. Furthermore, our study provide IS risk modules of SNPs, and related biological processes on transcriptome level. Obviously, future studies are needed to understand the underlying biological mechanism in gene-risk factors-IS/IS subtypes and the association between SNP-SNP interactions on IS/IS subtypes.

## Data Availability Statement

SNP data is available in the FigShare database^3^, 10.6084/m9.figshare.11993916.

## Ethics Statement

The research was approved by the ethics committee of 2nd Affiliated Hospital of Harbin Medical University.

## Author Contributions

XL drafted the manuscript, performed the cases collection and participated in the data analysis. WS participated in the data analysis and cases collection. RZ performed the cases collection and participated in the study design. SZ and WH directed the mediated result analysis. YW performed the MDR result analysis. RL and YF participated in statistical analyses. LS and JT designed and performed the experiments.

## Conflict of Interest

The authors declare that the research was conducted in the absence of any commercial or financial relationships that could be construed as a potential conflict of interest.

## Footnotes

1 http://www.biowing.com.cn

2 https://sourceforge.net/projects/mdr/

3 https://figshare.com/
