# Supplementary material for: Integrate Molecular Phenome and Polygenic Interaction to Detect the Genetic Risk of Ischemic Stroke
Source: Front Cell Dev Biol. 2020 Jun 24;8:453. doi: 10.3389/fcell.2020.00453 (PMC7326764; doi:10.3389/fcell.2020.00453)
Supplement: Supplementary file 1 [file Table_1.DOCX]

**Table 1 The characteristics of study subjects in the analysis of ischemic stroke**

| rs10887800 | AA | AG | GG | *p* | AA+AG | *p*’ | GG+AG | *p*’’ |
| --- | --- | --- | --- | --- | --- | --- | --- | --- |
| Age (year) | 55.1±9.7 | 54.7±9.3 | 55.6±10.2 | 0.9043 | 54.8±9.5 | 0.7437 | 55±9.6 | 0.6929 |
| Male n (%) | 91(37.4) | 181(35.9) | 95(37.5) | 0.873 | 272(36.4) | 0.8035 | 276(36.5) | 0.8401 |
| BMI (kg/m^2^) | 23.8±3.2 | 23.9±3.6 | 23.7±3 | 0.7052 | 23.9±3.4 | 0.5204 | 23.9±3.4 | 0.7237 |
| Systolic BP (mmHg) | 129.2±25.5 | 125±20 | 125.6±22.8 | 0.4857 | 126.4±22 | 0.9488 | 125.2±21 | 0.256 |
| Diastolic BP (mmHg) | 83±11.4 | 82±10.2 | 82.1±10.8 | 0.626 | 82.3±10.6 | 0.6089 | 82±10.4 | 0.5584 |
| Glu (mmol/l) | 6.2±4 | 5.5±1.7 | 5.5±2.1 | 0.2355 | 5.7±2.7 | 0.3361 | 5.5±1.8 | 0.0915 |
| TC (mmol/l) | 4.7±0.8 | 4.6±0.9 | 4.7±1 | 0.5787 | 4.7±0.8 | 0.3333 | 4.6±0.9 | 0.8727 |
| TG (mmol/l) | 1.3±1 | 1.3±1 | 1.3±1 | 0.3858 | 1.3±1 | 0.3726 | 1.3±1 | 0.5896 |
| HDL (mmol/l) | 1.4±0.3 | 1.4±0.3 | 1.4±0.5 | 0.454 | 1.4±0.3 | 0.3512 | 1.4±0.4 | 0.8075 |
| LDL (mmol/l) | 2.7±0.7 | 2.8±0.6 | 2.7±0.7 | 0.4453 | 2.8±0.6 | 0.208 | 2.7±0.6 | 0.7649 |
| Smoking n (%) | 98(40.3) | 175(34.7) | 92(36.4) | 0.3285 | 273(36.5) | 1 | 267(35.3) | 0.1775 |
| Drinking n (%) | 85(35.1) | 156(31.1) | 77(30.6) | 0.4668 | 241(32.4) | 0.6438 | 233(30.9) | 0.2516 |
| Atherothrombosis, n (%) | 20(15.4) | 40(13.6) | 21(13.8) | 0.8792 | 60(14.1) | 1 | 61(13.6) | 0.7198 |
| Lacunar, n (%) | 79(41.8) | 153(37.5) | 80(37.9) | 0.5869 | 232(38.9) | 0.8725 | 233(37.6) | 0.3461 |
| Combination, n (%) | 33(23.1) | 51(16.7) | 21(13.8) | 0.0972 | 84(18.7) | 0.2115 | 72(15.7) | 0.0579 |
| rs2576178 | CC | CT | TT | p | CC+CT | P’ | TT+CT | P’’ |
| Age (year) | 55.5±9.8 | 54.9±9.8 | 54.5±8.7 | 0.8633 | 55.1±9.8 | 0.8465 | 54.8±9.5 | 0.5882 |
| Female, n (%) | 98(32.7) | 193(38.6) | 75(38.9) | 0.197 | 291(36.4) | 0.576 | 268(38.7) | 0.0837 |
| BMI (kg/m2) | 23.7±3.3 | 23.9±3.4 | 23.8±3.4 | 0.7545 | 23.9±3.3 | 0.8233 | 23.9±3.4 | 0.5359 |
| Systolic BP (mmHg) | 126±21.2 | 126.2±22.2 | 126.6±24.3 | 0.1995 | 126.1±21.8 | 0.084 | 126.3±22.7 | 0.8714 |
| Diastolic BP (mmHg) | 81.8±10.2 | 82.4±10.9 | 82.7±10.9 | 0.7425 | 82.2±10.6 | 0.5114 | 82.5±10.9 | 0.5489 |
| Glu (mmol/l) | 5.7±3.2 | 5.5±1.9 | 6.0±3.0 | 0.4437 | 5.6±2.5 | 0.2493 | 5.6±2.2 | 0.9838 |
| TC (mmol/l) | 4.7±0.8 | 4.6±0.9 | 4.8±1.1 | 0.4988 | 4.6±0.8 | 0.9248 | 4.7±0.9 | 0.4859 |
| TG (mmol/l) | 1.3±1.2 | 1.3±0.8 | 1.4±1.2 | 0.31 | 1.3±0.9 | 0.3025 | 1.3±0.9 | 0.6543 |
| HDL (mmol/l) | 1.4±0.4 | 1.4±0.3 | 1.4±0.3 | 0.8517 | 1.4±0.3 | 0.7891 | 1.4±0.3 | 0.5764 |
| LDL (mmol/l) | 2.7±0.6 | 2.7±0.6 | 2.8±0.7 | 0.2684 | 2.7±0.6 | 0.9154 | 2.8±0.6 | 0.1324 |
| Smoking, n (%) | 104(34.7) | 187(37.4) | 70(36.3) | 0.7385 | 291(36.4) | 1 | 257(37.1) | 0.512 |
| Drinking, n (%) | 100(33.6) | 166(33.3) | 49(25.4) | 0.0994 | 266(33.4) | **0.0392** | 215(31.1) | 0.4951 |
| Atherothrombosis, n (%) | 25(8.4) | 41(8.2) | 14(7.3) | 0.9909 | 66(8.3) | 1 | 55(8) | 1 |
| Lacunar, n (%) | 97(32.7) | 143(28.8) | 71(36.8) | 0.0963 | 240(30.2) | 0.0547 | 214(31) | 0.9921 |
| Combination, n (%) | 23(7.7) | 57(11.5) | 24(12.4) | 0.1296 | 80(10.1) | 0.1922 | 81(11.7) | 0.0954 |
| rs2296545 | CC | CG | GG | *p* | CC+CG | *p*’ | GG+CG | *p*’’ |
| Age (year) | 55.3±9.6 | 54.8±9.9 | 54.9±8.9 | 0.6785 | 55±9.8 | 0.8753 | 54.8±9.7 | 0.3822 |
| Male n (%) | 121(35.2) | 180(37.7) | 64(37) | 0.7636 | 301(36.6) | 0.9948 | 244(37.5) | 0.5165 |
| BMI (kg/m^2^) | 23.7±3.2 | 23.9±3.4 | 24±3.4 | 0.7292 | 23.8±3.3 | 0.6931 | 23.9±3.4 | 0.4366 |
| Systolic BP (mmHg) | 126.5±21.7 | 126±21.8 | 126±24.4 | 0.3391 | 126.2±21.8 | 0.2396 | 126±22.5 | 0.699 |
| Diastolic BP (mmHg) | 82.2±10.3 | 82.3±10.9 | 82.4±10.9 | 0.9795 | 82.3±10.6 | 0.8834 | 82.3±10.9 | 0.8552 |
| Glu (mmol/l) | 5.7±3.2 | 5.6±2 | 5.8±2.5 | 0.8436 | 5.6±2.6 | 0.8779 | 5.6±2.2 | 0.5628 |
| TC (mmol/l) | 4.7±0.8 | 4.6±0.9 | 4.7±1 | 0.654 | 4.6±0.9 | 0.6707 | 4.7±0.9 | 0.3933 |
| TG (mmol/l) | 1.3±1 | 1.3±0.8 | 1.4±1.2 | 0.9973 | 1.3±0.9 | 0.9414 | 1.3±1 | 0.98 |
| HDL (mmol/l) | 1.4±0.4 | 1.4±0.3 | 1.4±0.3 | 0.2536 | 1.4±0.3 | 0.8287 | 1.4±0.3 | 0.1217 |
| LDL (mmol/l) | 2.7±0.6 | 2.7±0.6 | 2.8±0.7 | 0.5824 | 2.7±0.6 | 0.5558 | 2.8±0.6 | 0.3138 |
| Smoking n (%) | 109(31.7) | 190(39.7) | 64(37) | 0.0597 | 299(36.4) | 0.9466 | 254(39) | **0.0267** |
| Drinking n (%) | 108(31.6) | 164(34.5) | 45(26) | 0.1226 | 272(33.3) | 0.0775 | 209(32.2) | 0.8976 |
| Atherothrombosis, n (%) | 28(13.9) | 40(14.2) | 11(12.2) | 0.8897 | 68(14.1) | 0.7623 | 51(13.7) | 1 |
| Lacunar, n (%) | 107(38.1) | 138(36.4) | 66(45.5) | 0.1554 | 245(37.1) | 0.0741 | 204(38.9) | 0.8721 |
| Combination, n (%) | 32(15.5) | 56(18.9) | 17(17.7) | 0.6279 | 88(17.5) | 1 | 73(18.6) | 0.4141 |
| rs4073259 | AA | AG | GG | *p* | AA+AG | *p*’ | GG+AG | *p*’’ |
| Age (year) | 55.6±10 | 55.2±9.5 | 53.6±9.1 | 0.0767 | 55.4±9.7 | **0.024** | 54.7±9.4 | 0.3548 |
| Male n (%) | 105(35) | 180(37.9) | 79(36.4) | 0.7142 | 285(36.8) | 0.9841 | 259(37.4) | 0.5112 |
| BMI (kg/m^2^) | 23.7±3.4 | 23.8±3 | 24.2±3.9 | 0.528 | 23.8±3.2 | 0.2588 | 23.9±3.3 | 0.6187 |
| Systolic BP (mmHg) | 125±20.4 | 127±22.5 | 125.5±24.2 | 0.5486 | 126.2±21.7 | 0.2878 | 126.6±23 | 0.5096 |
| Diastolic BP (mmHg) | 81.2±9.2 | 82.9±11.4 | 82.4±10.8 | 0.2778 | 82.2±10.7 | 0.24 | 82.7±11.2 | 0.1579 |
| Glu (mmol/l) | 5.4±1.7 | 5.8±3.1 | 5.6±2.3 | 0.1103 | 5.6±2.6 | 0.9273 | 5.7±2.9 | **0.0375** |
| TC (mmol/l) | 4.6±0.9 | 4.7±0.9 | 4.6±0.8 | 0.8872 | 4.7±0.9 | 0.6588 | 4.7±0.8 | 0.629 |
| TG (mmol/l) | 1.3±1 | 1.3±1 | 1.2±0.8 | 0.8265 | 1.3±1 | 0.6263 | 1.3±1 | 0.7671 |
| HDL (mmol/l) | 1.3±0.3 | 1.4±0.3 | 1.5±0.5 | **0.0013** | 1.4±0.3 | 0.1141 | 1.4±0.4 | **0.0003** |
| LDL (mmol/l) | 2.7±0.7 | 2.8±0.6 | 2.7±0.5 | 0.7694 | 2.7±0.6 | 0.7636 | 2.8±0.6 | 0.4694 |
| Smoking n (%) | 105(35) | 174(36.6) | 83(38.2) | 0.7478 | 279(36) | 0.5972 | 257(37.1) | 0.5681 |
| Drinking n (%) | 86(28.7) | 159(33.6) | 72(33.5) | 0.3148 | 245(31.7) | 0.6776 | 231(33.6) | 0.1482 |
| Atherothrombosis, n (%) | 22(12.4) | 40(14.6) | 16(13) | 0.7883 | 62(13.7) | 0.9493 | 56(14.1) | 0.6822 |
| Lacunar, n (%) | 94(37.8) | 145(38.3) | 69(39.2) | 0.9546 | 239(38.1) | 0.8501 | 214(38.6) | 0.8892 |
| Combination, n (%) | 27(14.8) | 52(18.2) | 25(18.9) | 0.556 | 79(16.9) | 0.6732 | 77(18.4) | 0.3424 |
| rs17286604 | CC | CT | TT | *p* |  |  |  |  |
| Age (year) | 54.8±9.6 | 59.4±10.3 | - | 0.1769 |  |  |  |  |
| Male n (%) | 357(37) | 9(30) | - | 0.5578 |  |  |  |  |
| BMI (kg/m^2^) | 23.9±3.4 | 23.9±2.9 | - | 0.9783 |  |  |  |  |
| Systolic BP (mmHg) | 126.1±22.2 | 128.3±20.1 | - | 0.5975 |  |  |  |  |
| Diastolic BP (mmHg) | 82.3±10.7 | 83±8.5 | - | 0.8473 |  |  |  |  |
| Glu (mmol/l) | 5.6±2.4 | 6±4.2 | - | 0.8021 |  |  |  |  |
| TC (mmol/l) | 4.7±0.9 | 4.5±0.8 | - | 0.716 |  |  |  |  |
| TG (mmol/l) | 1.3±1 | 1.2±0.6 | - | 0.6578 |  |  |  |  |
| HDL (mmol/l) | 1.4±0.3 | 1.4±0.3 | - | 0.3872 |  |  |  |  |
| LDL (mmol/l) | 2.7±0.6 | 2.7±0.6 | - | 0.9758 |  |  |  |  |
| Smoking n (%) | 350(36.2) | 12(40) | - | 0.8182 |  |  |  |  |
| Drinking n (%) | 306(31.8) | 10(33.3) | - | 1 |  |  |  |  |
| Atherothrombosis, n (%) | 79(14.1) | 1(6.7) | - | 0.7065 |  |  |  |  |
| Lacunar, n (%) | 301(38.5) | 11(44) | - | 0.7315 |  |  |  |  |
| Combination, n (%) | 100(17.2) | 4(22.2) | - | 0.5337 |  |  |  |  |
| rs7620580 | AA | AG | GG | *p* | AA+AG | *p*’ | GG+AG | *p*’’ |
| Age (year) | 54.8±9.4 | 55.1±9.7 | 56.8±11.4 | 0.5021 | 54.9±9.5 | 0.2446 | 55.3±9.9 | 0.7834 |
| Male n (%) | 221(38) | 122(33.5) | 23(46) | 0.1457 | 343(36.3) | 0.2142 | 145(35) | 0.3764 |
| BMI (kg/m^2^) | 23.7±3.4 | 24.1±3.3 | 23.8±2.9 | 0.2716 | 23.9±3.4 | 0.7994 | 24.1±3.3 | 0.1322 |
| Systolic BP (mmHg) | 126.4±23.3 | 126.2±20.4 | 123.2±20.3 | 0.3855 | 126.3±22.3 | 0.1752 | 125.9±20.4 | 0.8691 |
| Diastolic BP (mmHg) | 82.3±10.7 | 82.2±10.3 | 82.1±11.7 | 0.862 | 82.3±10.5 | 0.8164 | 82.2±10.4 | 0.6792 |
| Glu (mmol/l) | 5.6±2.1 | 5.7±3.1 | 5.4±1.3 | 0.8904 | 5.6±2.5 | 0.9738 | 5.7±3 | 0.6317 |
| TC (mmol/l) | 4.7±0.9 | 4.6±0.8 | 4.4±0.8 | 0.417 | 4.7±0.9 | 0.237 | 4.6±0.8 | 0.2453 |
| TG (mmol/l) | 1.3±1 | 1.3±1 | 1.1±0.4 | 0.6115 | 1.3±1 | 0.4117 | 1.3±0.9 | 0.442 |
| HDL (mmol/l) | 1.4±0.4 | 1.4±0.3 | 1.4±0.3 | 0.2444 | 1.4±0.3 | 0.5326 | 1.4±0.3 | 0.0912 |
| LDL (mmol/l) | 2.8±0.6 | 2.7±0.6 | 2.5±0.7 | 0.5906 | 2.7±0.6 | 0.332 | 2.7±0.6 | 0.9728 |
| Smoking n (%) | 199(34.2) | 144(39.6) | 19(38) | 0.2403 | 343(36.3) | 0.9213 | 163(39.4) | 0.1078 |
| Drinking n (%) | 173(29.8) | 129(35.7) | 14(28) | 0.1353 | 302(32.1) | 0.6566 | 143(34.8) | 0.1093 |
| Atherothrombosis, n (%) | 41(12.1) | 35(16.7) | 4(14.8) | 0.303 | 76(13.9) | 0.7808 | 39(16.5) | 0.1696 |
| Lacunar, n (%) | 182(38) | 115(39.8) | 15(39.5) | 0.8803 | 297(38.7) | 1 | 130(39.8) | 0.6672 |
| Combination, n (%) | 60(16.8) | 36(17.1) | 8(25.8) | 0.4444 | 96(16.9) | 0.3048 | 44(18.3) | 0.727 |
| rs11712619 | CC | CT | TT | *p* |  |  |  |  |
| Age (year) | 54.9±9.6 | 58.9±7.5 | - | 0.1964 |  |  |  |  |
| Male n (%) | 360(36.8) | 6(31.6) | - | 0.8169 |  |  |  |  |
| BMI (kg/m^2^) | 23.9±3.3 | 23.6±3.1 | - | 0.7174 |  |  |  |  |
| Systolic BP (mmHg) | 126.1±22.2 | 132.1±18.5 | - | 0.686 |  |  |  |  |
| Diastolic BP (mmHg) | 82.2±10.6 | 84.1±8.1 | - | 0.5128 |  |  |  |  |
| Glu (mmol/l) | 5.6±2.4 | 6.8±5.3 | - | 0.5672 |  |  |  |  |
| TC (mmol/l) | 4.7±0.9 | 4.6±0.9 | - | 0.5578 |  |  |  |  |
| TG (mmol/l) | 1.3±1 | 1.7±1.9 | - | 0.6118 |  |  |  |  |
| HDL (mmol/l) | 1.4±0.3 | 1.2±0.2 | - | **0.0032** |  |  |  |  |
| LDL (mmol/l) | 2.7±0.6 | 2.7±0.6 | - | 0.8511 |  |  |  |  |
| Smoking n (%) | 353(36.1) | 9(47.4) | - | 0.4426 |  |  |  |  |
| Drinking n (%) | 310(31.9) | 6(31.6) | - | 1 |  |  |  |  |
| Atherothrombosis, n (%) | 80(14.1) | 0(0) | - | 1 |  |  |  |  |
| Lacunar, n (%) | 301(38.1) | 11(64.7) | - | **0.0486** |  |  |  |  |
| Combination, n (%) | 102(17.3) | 2(25) | - | 0.6332 |  |  |  |  |
| rs6438833 | AA | AT | TT | *p* | AA+AT | *p*’ | TT+AT | *p*’’ |
| Age (year) | 49.8±7 | 53.8±9 | 55.3±9.7 | **0.0485** | 53.6±9 | 0.1476 | 55±9.6 | **0.0471** |
| Male n (%) | 1(16.7) | 49(32.7) | 316(37.6) | 0.3734 | 50(32.1) | 0.2171 | 365(36.9) | 0.4232 |
| BMI (kg/m^2^) | 27.4±3.7 | 24±2.9 | 23.8±3.4 | 0.1592 | 24.2±3 | 0.2946 | 23.8±3.3 | 0.0973 |
| Systolic BP (mmHg) | 116±11.4 | 128.8±25.1 | 125.8±21.6 | **0.006** | 128.2±24.7 | 0.606 | 126.3±22.2 | **0.0099** |
| Diastolic BP (mmHg) | 78±8.4 | 83.2±10.6 | 82.1±10.6 | 0.1489 | 83±10.6 | 0.4853 | 82.3±10.6 | 0.1091 |
| Glu (mmol/l) | 5.5±0.6 | 5.7±3.8 | 5.6±2.2 | 0.167 | 5.7±3.8 | 0.8288 | 5.6±2.5 | 0.0857 |
| TC (mmol/l) | 4.9±0.8 | 4.7±1 | 4.7±0.9 | 0.5416 | 4.7±1 | 0.287 | 4.7±0.9 | 0.8301 |
| TG (mmol/l) | 1.1±0.4 | 1.2±0.7 | 1.3±1 | 0.1266 | 1.2±0.6 | 0.1293 | 1.3±1 | 0.122 |
| HDL (mmol/l) | 1.4±0.2 | 1.4±0.3 | 1.4±0.4 | 0.5802 | 1.4±0.3 | 0.2874 | 1.4±0.3 | 0.9182 |
| LDL (mmol/l) | 3.1±0.5 | 2.8±0.5 | 2.7±0.6 | 0.2861 | 2.8±0.5 | 0.4571 | 2.7±0.6 | 0.1746 |
| Smoking n (%) | 3(50) | 63(42) | 296(35.2) | 0.2123 | 66(42.3) | 0.1107 | 359(36.3) | 0.6738 |
| Drinking n (%) | 3(50) | 51(34) | 262(31.3) | 0.4678 | 54(34.6) | 0.4761 | 313(31.7) | 0.3898 |
| Atherothrombosis, n (%) | 1(20) | 9(9.8) | 70(14.7) | 0.3313 | 10(10.3) | 0.3316 | 79(13.9) | 0.5292 |
| Lacunar, n (%) | 0(0) | 39(32) | 273(40.1) | 0.07 | 39(31) | 0.0648 | 312(38.9) | 0.1626 |
| Combination, n (%) | 1(20) | 19(18.6) | 84(17.1) | 0.8217 | 20(18.7) | 0.8019 | 103(17.4) | 1 |
| rs243330 | AA | AG | GG | *p* | AA+AG | p’ | GG+AG | p’’ |
| Age (year) | 55.4±9.8 | 54.1±9.3 | 56±8.7 | 0.1812 | 54.9±9.6 | 0.3741 | 54.2±9.2 | 0.1683 |
| Male n (%) | 216(36.4) | 128(36.8) | 15(39.5) | 0.9259 | 344(36.5) | 0.8422 | 143(37) | 0.8816 |
| BMI (kg/m^2^) | 23.9±3 | 23.8±3.8 | 24.5±4 | 0.7245 | 23.9±3.3 | 0.4357 | 23.9±3.8 | 0.7331 |
| Systolic BP (mmHg) | 127.5±23.1 | 124±20.6 | 124.8±21.5 | 0.3384 | 126.2±22.3 | 0.9702 | 124.1±20.7 | 0.1471 |
| Diastolic BP (mmHg) | 82.8±11 | 81.8±10 | 80.3±9.3 | 0.6142 | 82.4±10.6 | 0.8897 | 81.7±9.9 | 0.325 |
| Glu (mmol/l) | 5.7±2.6 | 5.6±2.4 | 5±0.6 | 0.9614 | 5.6±2.5 | 0.78 | 5.6±2.3 | 0.9395 |
| TC (mmol/l) | 4.6±0.8 | 4.7±0.9 | 4.3±0.8 | 0.1825 | 4.7±0.9 | 0.0671 | 4.7±0.9 | 0.3631 |
| TG (mmol/l) | 1.3±1 | 1.3±0.9 | 1.2±0.9 | 0.6154 | 1.3±1 | 0.6138 | 1.3±0.9 | 0.5278 |
| HDL (mmol/l) | 1.4±0.3 | 1.4±0.4 | 1.4±0.3 | 0.1161 | 1.4±0.3 | 0.293 | 1.4±0.4 | 0.0801 |
| LDL (mmol/l) | 2.7±0.6 | 2.8±0.6 | 2.5±0.8 | 0.1992 | 2.7±0.6 | 0.0801 | 2.7±0.6 | 0.7883 |
| Smoking n (%) | 224(37.7) | 121(34.8) | 14(36.8) | 0.6643 | 345(36.6) | 1 | 135(35) | 0.4232 |
| Drinking n (%) | 193(32.5) | 112(32.5) | 7(18.4) | 0.1885 | 305(32.5) | 0.0991 | 119(31.1) | 0.68 |
| Atherothrombosis, n (%) | 50(14.9) | 23(11.2) | 6(26.1) | 0.1113 | 73(13.5) | 0.1161 | 29(12.7) | 0.5336 |
| Lacunar, n (%) | 194(40.4) | 102(35.8) | 13(43.3) | 0.392 | 296(38.7) | 0.7485 | 115(36.5) | 0.3023 |
| Combination, n (%) | 59(17.1) | 39(17.6) | 2(10.5) | 0.8755 | 98(17.3) | 0.7551 | 41(17) | 1 |
| rs161818 | AA | AG | GG | *p* | AA+AG | *p*’ | GG+AG | *p*’’ |
| Age (year) | 54.1±9.5 | 55±9.8 | 55.4±9.3 | 0.4825 | 54.7±9.7 | 0.3372 | 55.2±9.6 | 0.3125 |
| Male n (%) | 81(39.9) | 169(35.8) | 109(35.7) | 0.5546 | 250(37) | 0.7495 | 278(35.8) | 0.3155 |
| BMI (kg/m^2^) | 23.9±3.6 | 23.8±3.3 | 23.9±3.2 | 0.9321 | 23.9±3.4 | 0.9976 | 23.9±3.3 | 0.7283 |
| Systolic BP (mmHg) | 122.5±21.2 | 126.9±23.4 | 127.6±20.9 | **0.0377** | 125.5±22.8 | 0.1171 | 127.1±22.4 | **0.0152** |
| Diastolic BP (mmHg) | 80.9±8.5 | 82.6±11.3 | 83±10.6 | 0.0646 | 82.1±10.6 | 0.2087 | 82.7±11 | **0.0242** |
| Glu (mmol/l) | 5.4±1.2 | 5.7±2.4 | 5.7±3.2 | 0.8315 | 5.6±2.1 | 0.9547 | 5.7±2.7 | 0.5583 |
| TC (mmol/l) | 4.7±1 | 4.6±0.8 | 4.7±0.9 | 0.6725 | 4.7±0.9 | 0.3745 | 4.7±0.8 | 0.591 |
| TG (mmol/l) | 1.3±0.9 | 1.3±1 | 1.3±1 | 0.801 | 1.3±1 | 0.5246 | 1.3±1 | 0.735 |
| HDL (mmol/l) | 1.4±0.3 | 1.4±0.3 | 1.4±0.4 | 0.5688 | 1.4±0.3 | 0.4171 | 1.4±0.4 | 0.3727 |
| LDL (mmol/l) | 2.8±0.7 | 2.7±0.6 | 2.7±0.6 | 0.4084 | 2.7±0.6 | 0.5329 | 2.7±0.6 | 0.1827 |
| Smoking n (%) | 71(35) | 181(38.3) | 107(35.1) | 0.5616 | 252(37.3) | 0.5447 | 288(37.1) | 0.6394 |
| Drinking n (%) | 67(33) | 148(31.6) | 97(31.9) | 0.9336 | 215(32) | 1 | 245(31.7) | 0.7859 |
| Atherothrombosis, n (%) | 16(12.7) | 43(15.8) | 20(12) | 0.4968 | 59(14.8) | 0.4704 | 63(14.4) | 0.7446 |
| Lacunar, n (%) | 61(35.7) | 147(39) | 101(40.9) | 0.5592 | 208(38) | 0.4796 | 248(39.7) | 0.3794 |
| Combination, n (%) | 16(12.7) | 50(17.9) | 34(18.9) | 0.3255 | 66(16.3) | 0.5077 | 84(18.3) | 0.1813 |
| rs33932899 | CC | CG | GG | p | CC+CG | *p*’ | GG+CG | *p*’’ |
| Age (year) | 54.8±8.3 | 54±9.2 | 55.5±9.8 | 0.1481 | 54±9.1 | 0.144 | 55±9.6 | 0.3921 |
| Male n (%) | 15(38.5) | 120(37.5) | 224(36.1) | 0.885 | 135(37.6) | 0.6809 | 344(36.6) | 0.9423 |
| BMI (kg/m^2^) | 24.5±4.1 | 23.7±3.8 | 23.9±3.1 | 0.7003 | 23.8±3.8 | 0.7417 | 23.9±3.3 | 0.5008 |
| Systolic BP (mmHg) | 123±21.2 | 123.9±21.1 | 127.5±22.8 | 0.1804 | 123.8±21.1 | 0.063 | 126.3±22.3 | 0.7873 |
| Diastolic BP (mmHg) | 81±9.9 | 81.5±10.2 | 82.8±10.8 | 0.4543 | 81.5±10.2 | 0.2448 | 82.4±10.6 | 0.8815 |
| Glu (mmol/l) | 5±0.6 | 5.6±2.5 | 5.6±2.6 | 0.8222 | 5.6±2.4 | 0.8124 | 5.6±2.5 | 0.5347 |
| TC (mmol/l) | 4.4±0.8 | 4.7±0.9 | 4.7±0.9 | 0.5136 | 4.7±0.9 | 0.2648 | 4.7±0.9 | 0.4131 |
| TG (mmol/l) | 1.2±1 | 1.3±0.9 | 1.3±1 | 0.2502 | 1.3±0.9 | 0.4347 | 1.3±1 | 0.2863 |
| HDL (mmol/l) | 1.4±0.3 | 1.4±0.4 | 1.4±0.3 | 0.0562 | 1.4±0.4 | 0.1545 | 1.4±0.3 | 0.0637 |
| LDL (mmol/l) | 2.5±0.8 | 2.7±0.6 | 2.7±0.6 | 0.4879 | 2.7±0.6 | 0.5024 | 2.7±0.6 | 0.2477 |
| Smoking n (%) | 14(35.9) | 111(34.7) | 234(37.7) | 0.6621 | 125(34.8) | 0.4081 | 345(36.7) | 1 |
| Drinking n (%) | 6(15.4) | 105(33.1) | 201(32.4) | 0.0749 | 111(31.2) | 0.7426 | 306(32.7) | **0.0365** |
| Atherothrombosis, n (%) | 6(27.3) | 23(11.8) | 50(14.4) | 0.1386 | 29(13.4) | 0.8337 | 73(13.4) | 0.1061 |
| Lacunar, n (%) | 14(46.7) | 90(34.4) | 205(40.8) | 0.1517 | 104(35.6) | 0.1746 | 295(38.6) | 0.4824 |
| Combination, n (%) | 3(15.8) | 34(16.5) | 63(17.5) | 0.9553 | 37(16.4) | 0.8397 | 97(17.1) | 1 |
| rs161810 | AA | AG | GG | p | AA+AG | *p*’ | GG+AG | *p*’’ |
| Age (year) | 54.1±9.5 | 55.1±9.9 | 55.3±9.2 | 0.4913 | 54.8±9.8 | 0.3942 | 55.2±9.6 | 0.2843 |
| Male n (%) | 80(39.2) | 168(36) | 111(35.9) | 0.6905 | 248(37) | 0.8089 | 279(36) | 0.4361 |
| BMI (kg/m^2^) | 23.9±3.6 | 23.8±3.3 | 24±3.2 | 0.8593 | 23.8±3.4 | 0.7982 | 23.9±3.3 | 0.7204 |
| Systolic BP (mmHg) | 122.5±21.1 | 126.6±23.5 | 127.9±20.9 | **0.03** | 125.4±22.8 | 0.0745 | 127.1±22.5 | **0.0149** |
| Diastolic BP (mmHg) | 80.9±8.5 | 82.5±11.4 | 83.1±10.6 | **0.0497** | 82±10.6 | 0.1392 | 82.7±11 | **0.0214** |
| Glu (mmol/l) | 5.4±1.2 | 5.7±2.4 | 5.7±3.2 | 0.8204 | 5.6±2.1 | 0.8396 | 5.7±2.7 | 0.5294 |
| TC (mmol/l) | 4.7±1 | 4.7±0.8 | 4.6±0.9 | 0.5426 | 4.7±0.9 | 0.2698 | 4.7±0.8 | 0.5973 |
| TG (mmol/l) | 1.3±1 | 1.3±1 | 1.3±1 | 0.6812 | 1.3±1 | 0.4553 | 1.3±1 | 0.5357 |
| HDL (mmol/l) | 1.4±0.3 | 1.4±0.3 | 1.4±0.4 | 0.4973 | 1.4±0.3 | 0.49 | 1.4±0.4 | 0.2633 |
| LDL (mmol/l) | 2.8±0.7 | 2.7±0.6 | 2.7±0.6 | 0.3452 | 2.7±0.6 | 0.2374 | 2.7±0.6 | 0.2153 |
| Smoking n (%) | 73(35.8) | 177(37.9) | 109(35.3) | 0.7289 | 250(37.3) | 0.598 | 286(36.9) | 0.8407 |
| Drinking n (%) | 69(33.8) | 143(30.8) | 100(32.5) | 0.7262 | 212(31.7) | 0.8778 | 243(31.5) | 0.579 |
| Atherothrombosis, n (%) | 16(12.8) | 41(15.1) | 22(13) | 0.7511 | 57(14.4) | 0.7646 | 63(14.3) | 0.775 |
| Lacunar, n (%) | 63(36.6) | 144(38.5) | 102(41) | 0.6555 | 207(37.9) | 0.4591 | 246(39.5) | 0.5535 |
| Combination, n (%) | 16(12.8) | 50(17.9) | 34(18.8) | 0.3484 | 66(16.3) | 0.5346 | 84(18.2) | 0.1953 |
| rs161827 | CC | CT | TT | p | CC+CT | *p*’ | TT+CT | *p*’’ |
| Age (year) | 55.4±9.2 | 55.1±9.9 | 54±9.5 | 0.5293 | 55.2±9.6 | 0.3793 | 54.8±9.8 | 0.338 |
| Male n (%) | 108(35.8) | 169(35.8) | 82(39.8) | 0.5679 | 277(35.8) | 0.326 | 251(37) | 0.7597 |
| BMI (kg/m^2^) | 23.9±3.2 | 23.8±3.4 | 23.9±3.6 | 0.8937 | 23.9±3.3 | 0.6398 | 23.9±3.4 | 0.9186 |
| Systolic BP (mmHg) | 127.3±20.6 | 127±23.6 | 122.5±21.1 | 0.0649 | 127.1±22.5 | **0.0272** | 125.7±22.9 | 0.1464 |
| Diastolic BP (mmHg) | 83±10.6 | 82.6±11.3 | 80.9±8.5 | 0.1135 | 82.7±11 | 0.0506 | 82.1±10.6 | 0.201 |
| Glu (mmol/l) | 5.7±3.2 | 5.7±2.4 | 5.4±1.2 | 0.8302 | 5.7±2.8 | 0.5458 | 5.6±2.1 | 0.8915 |
| TC (mmol/l) | 4.7±0.9 | 4.6±0.8 | 4.7±1 | 0.6805 | 4.7±0.8 | 0.7536 | 4.7±0.9 | 0.3886 |
| TG (mmol/l) | 1.3±1 | 1.3±1 | 1.3±0.9 | 0.7085 | 1.3±1 | 0.487 | 1.3±1 | 0.5392 |
| HDL (mmol/l) | 1.4±0.4 | 1.4±0.3 | 1.4±0.3 | 0.4753 | 1.4±0.4 | 0.2968 | 1.4±0.3 | 0.3638 |
| LDL (mmol/l) | 2.7±0.6 | 2.7±0.6 | 2.8±0.7 | 0.3922 | 2.7±0.6 | 0.1736 | 2.7±0.6 | 0.5145 |
| Smoking n (%) | 104(34.4) | 181(38.3) | 74(35.9) | 0.53 | 285(36.8) | 0.8754 | 255(37.6) | 0.3787 |
| Drinking n (%) | 97(32.2) | 146(31.1) | 69(33.5) | 0.8263 | 243(31.6) | 0.6561 | 215(31.9) | 0.967 |
| Atherothrombosis, n (%) | 20(12.1) | 43(15.7) | 16(12.7) | 0.5182 | 63(14.4) | 0.7446 | 59(14.8) | 0.4928 |
| Lacunar, n (%) | 99(40.6) | 145(38.6) | 65(37.1) | 0.7662 | 244(39.4) | 0.6583 | 210(38.1) | 0.5634 |
| Combination, n (%) | 34(19) | 51(18.1) | 15(12) | 0.2295 | 85(18.4) | 0.118 | 66(16.2) | 0.4813 |
| rs243327 | CC | CT | TT | *p* | CC+CT | *p*’ | TT+CT | *p*’’ |
| Age (year) | 55.5±9.8 | 54±9.2 | 56.2±8.9 | 0.1795 | 54.9±9.6 | 0.4494 | 54.2±9.2 | 0.1395 |
| Male n (%) | 214(36.2) | 131(37.3) | 14(37.8) | 0.9325 | 345(36.6) | 1 | 145(37.4) | 0.7634 |
| BMI (kg/m^2^) | 23.9±3 | 23.8±3.8 | 24.6±4.1 | 0.6876 | 23.9±3.3 | 0.3919 | 23.9±3.8 | 0.8727 |
| Systolic BP (mmHg) | 127.6±23.1 | 123.8±20.5 | 124.7±22.4 | 0.2255 | 126.2±22.3 | 0.8892 | 123.9±20.6 | 0.0979 |
| Diastolic BP (mmHg) | 82.8±11 | 81.7±10 | 80.3±9.8 | 0.4407 | 82.4±10.6 | 0.8377 | 81.6±9.9 | 0.2431 |
| Glu (mmol/l) | 5.7±2.6 | 5.6±2.4 | 5±0.6 | 0.8839 | 5.6±2.5 | 0.6855 | 5.6±2.3 | 0.8676 |
| TC (mmol/l) | 4.7±0.8 | 4.7±0.9 | 4.4±0.8 | 0.2379 | 4.7±0.9 | 0.0938 | 4.7±0.9 | 0.3315 |
| TG (mmol/l) | 1.3±1 | 1.3±0.9 | 1.2±1 | 0.4328 | 1.3±1 | 0.4473 | 1.3±0.9 | 0.4625 |
| HDL (mmol/l) | 1.4±0.3 | 1.4±0.4 | 1.4±0.3 | 0.064 | 1.4±0.3 | 0.1769 | 1.4±0.4 | 0.0644 |
| LDL (mmol/l) | 2.7±0.6 | 2.7±0.6 | 2.6±0.8 | 0.2233 | 2.7±0.6 | 0.0882 | 2.7±0.6 | 0.661 |
| Smoking n (%) | 223(37.7) | 121(34.5) | 14(37.8) | 0.5959 | 344(36.5) | 1 | 135(34.8) | 0.3865 |
| Drinking n (%) | 194(32.9) | 111(31.9) | 7(18.9) | 0.2099 | 305(32.5) | 0.1189 | 118(30.6) | 0.5092 |
| Atherothrombosis, n (%) | 49(14.8) | 23(10.9) | 6(28.6) | 0.0621 | 72(13.3) | 0.0562 | 29(12.5) | 0.5217 |
| Lacunar, n (%) | 195(40.8) | 100(34.7) | 14(48.3) | 0.1415 | 295(38.5) | 0.3872 | 114(36) | 0.1955 |
| Combination, n (%) | 59(17.3) | 39(17.2) | 2(11.8) | 0.955 | 98(17.2) | 0.7501 | 41(16.8) | 0.9754 |

BMI: body mass index; BP: blood pressure; Glu: glucose; TC: total cholesterol; TG: triglyceride; HDL: high density lipoprotein; LDL: low density lipoprotein; Atherothrombosis: Atherothrombosis ischemic stroke subtype; Lacunar: lacunar ischemic stroke subtype; Combination: combination ischemic stroke subtype.

*P*<0.05 are shown in bold
